# Variations in the Electrostatic Landscape of Class II Human Leukocyte Antigen Molecule Induced by Modifications in the Myelin Basic Protein Peptide: A Theoretical Approach

**DOI:** 10.1371/journal.pone.0004164

**Published:** 2009-01-09

**Authors:** William A. Agudelo, Johan F. Galindo, Marysol Ortiz, José L. Villaveces, Edgar E. Daza, Manuel E. Patarroyo

**Affiliations:** 1 Grupo de Biomatemáticas, Fundación Instituto de Inmunología de Colombia, Bogotá, Colombia; 2 Grupo de Química Teórica, Universidad Nacional de Colombia, Centro de Investigaciones en Sistemas complejos CEIBA, Bogotá, Colombia; 3 Grupo de Química Teórica, Universidad de los Andes, Centro de Investigaciones en Sistemas Complejos CEIBA, Bogotá, Colombia; Institute of Molecular and Cell Biology, Singapore

## Abstract

The receptor-ligand interactions involved in the formation of the complex between Class II Major Histocompatibility Complex molecules and antigenic peptides, which are essential for establishing an adaptive immunological response, were analyzed in the Class II Human Leukocyte Antigen (HLA) - Myelin Basic Protein (MBP) peptide complex (HLA-DRβ1*1501-MBP) using a multipolar molecular electrostatic potential approach. The Human Leukocyte Antigen - peptide complex system was divided into four pockets together with their respective peptide fragment and the corresponding occupying amino acid was replaced by each of the remaining 19 amino acids. Partial atomic charges were calculated by a quantum chemistry approach at the Hatree Fock/3-21*G level, to study the behavior of monopole, dipole and quadrupole electrostatic multipolar moments. Two types of electrostatic behavior were distinguished in the pockets' amino acids: “anchoring” located in Pocket 1 and 4, and “recognition” located in Pocket 4 and 7. According to variations in the electrostatic landscape, pockets were ordered as: Pocket 1>Pocket 9≫Pocket 4≈Pocket 7 which is in agreement with the binding ability reported for Class II Major Histocompatibility Complex pockets. In the same way, amino acids occupying the polymorphic positions β13R, β26F, β28D, β9W, β74A, β47F and β57D were shown to be key for this Receptor-Ligand interaction. The results show that the multipolar molecular electrostatic potential approach is appropriate for characterizing receptor-ligand interactions in the MHC–antigenic peptide complex, which could have potential implications for synthetic vaccine design.

## Introduction

In the last years, a vast body of information regarding the interaction of short synthetic peptides (∼20-mer long) derived from the amino acid sequences of *Plasmodium falciparum* proteins (the most lethal and prevalent agent of human malaria responsible for 500 million cases per year, of which 3 million result in death [1]); with their corresponding host cell receptor have been obtained [2]–[8].

One of the most important steps for developing a successful immune response is the formation of the appropriate complex between Major Histocompatibility Complex (MHC) molecule and antigenic peptides, with the subsequent recognition and reading of this complex by the T cell receptor (TCR) molecules, which determines the generation of an appropriate immune response against the pathogen.

The MHC Class II molecules, responsible for the presentation of some type of these antigens, are membrane glycoproteins formed by a highly conserved amino acid chain molecule of 34 kDa, called *α chain*, and a highly polymorphic protein of 28 kDa called *β chain*. These two chains are associated by non-covalent interactions forming a macromolecular complex where three distinctive regions are recognized: an intracellular, a transmembrane and an extracellular region, the latter comprising about two thirds of the molecule. The most distal portion of the extracellular region contains a groove or through named Peptide Binding Region (PBR), formed by two flanking α helices and a β sheets floor, where peptides anchor to enable their presentation to the TCR.

Due to the degree of genetic polymorphism of MHC-II molecules codified in the so called **H**uman **L**eukocyte **A**ntigen **D**-**R**elated genetic region (HLA-DR) [9], 16 alleles named HLA-DRβ1*01 to HLA-DRβ1*16 with more than 300 variants have been identified, and serologically, functionally, evolutively and molecular classified [10], [11]. These alleles are grouped into five groups or haplotypes by following the same principles: HLA-DR1 including HLA-DRβ1*101,10,103,104 alleles; HLA-DR51 including HLA-DRβ1*15 and 16 alleles; HLA-DR52 including HLA-DRβ1*03,11,12,13 and 14 alleles; HLA-DR8 including only HLA-DRβ1*08 and HLA-DR53 including HLA-DRβ1*04,07,09 alleles [12].

**Figure 1:**
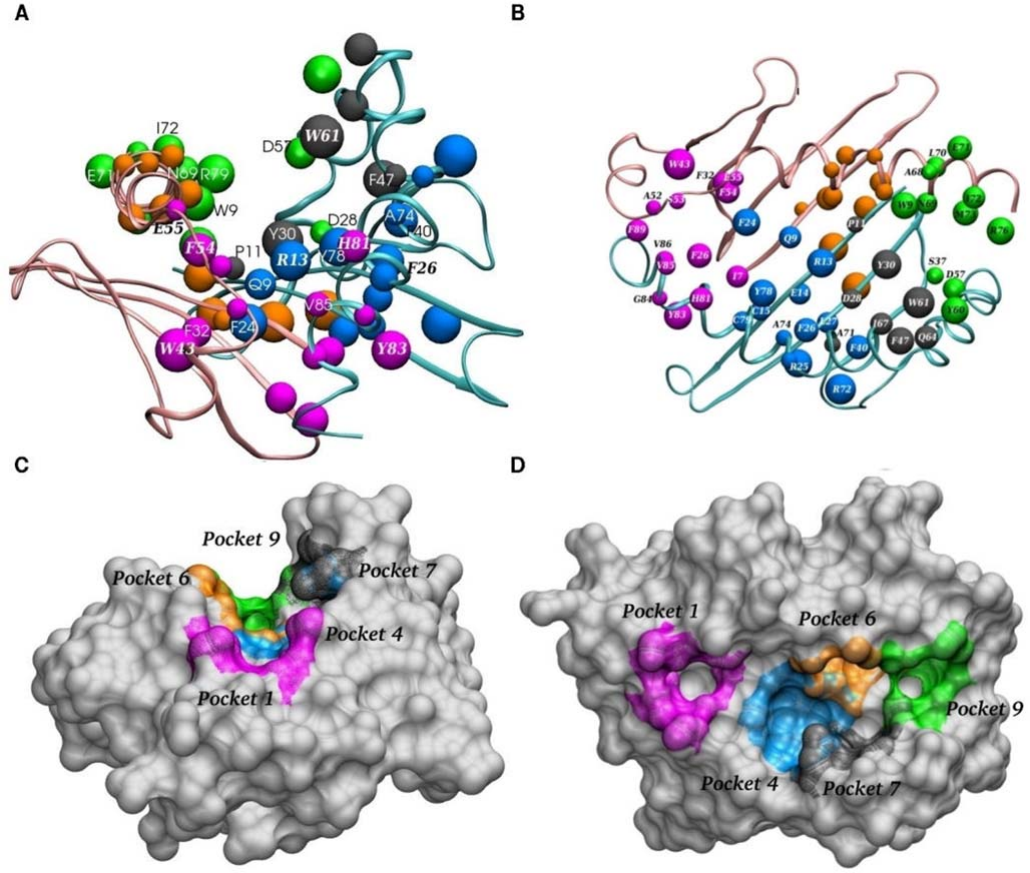
The Peptide Binding Region (PBR) of the Major Histocompatibility Complex Class II. HLA-DRβ1*1501 molecule showing the α-chain as a pink ribbon and the β-chain as a light blue ribbon. (A) Frontal view and (B) top view. The amino acids defining each pocket are represented as balls according to their size in a different color: Pocket 1 (magenta), Pocket 4 (dark blue), Pocket 7 (gray) and Pocket 9 (green). Maximal Speed Molecular Surface representation (www.scripps.edu/~sanner/html/msms_home.html) [37] (C) Frontal view evidencing the trough of the PBR and (D) Top view showing the deep pockets P1 and P9 whereas Pocket 4, 6 and 7, more superficial, lye towards the walls of the groove. These figures were made using the VMD software [24].

Elegant and seminal crystallographic studies with these purified molecules have shown that interaction with the antigenic peptide, occurs in specific regions located in the PBR called Pockets (represented by different color in Fig. 1A–D), which are assumed to be relatively independent [13], [14], therefore allowing each pocket-peptide interaction to be studied separately (see Table 1). Even though this is clearly a simplification based on experimental results, it is convenient for the purpose of this study since we are simply interested in identifying patterns of interaction. In the canonical binding pattern, the peptide amino acid's side chains must fit into two deep pockets formed by amino acids from α as well as β chains named Pocket 1 (P1) and Pocket 9 (P9) (Fig. 1); and two more shallow pockets named Pocket 4 (P4), formed preferentially by β chain amino acids, and Pocket 7 (P7), formed mainly by β chain amino acids. In classical HLA-DR molecules, there is also an additional Pocket named Pocket 6 (P6), which is formed mainly by amino acids of the α chain, but is not relevant for this allele as it will be discussed later on.

**Table 1:** Amino acids defining each pocket and peptide sequence considered in the electrostatic analysis.

**Pocket 1**	α-Chain	I7 F24 ***D25*** F26 F32 ***H33*** W43 A52 S53 F54 E55
	β−Chain	H81 Y83 G84 V85 V86 F89 T90 V91
	Peptide	E85 N86 P87 V88 *X89* H90
**Pocket 4**	α-Chain	Q9 A10 E11 F24 ***D25 D27*** N62
	β−Chain	R13 E14 C15 R25 F26 L27 D28 F40 Q70 A71 R72 A73 A74 Y78 C79
	Peptide	F91 *X92* K93
**Pocket 7**	α-Chain	V65 ***D66 K67*** N69
	β−Chain	P11 R13 Y30 D28 V38 F47 W61 Q64 I67 A71
	Peptide	N94 *X95* V96
**Pocket 9**	α-Chain	***D66*** A68 N69 L70 E71 I72 M73 R76
	β−Chain	W9 ***R29*** Y30 S37 V38 D57 ***E59*** Y60 W61
	Peptide	V96 *X97* P98 R99

The amino acids in the α and β chains are shown for each pocket. The underlined amino acid corresponds to the occupying amino acid while bold-type amino acids were added to the system whenever necessary for assuring the neutral charge of the system for computational reasons.

Given that these amino acids provide a proper environment for the peptide's “occupying amino acid” to fit properly into each of the PBR pockets [10], [15], [16], the present study seeks to find the determining factors ruling these interactions, under the hypotheses that their nature is electrostatic and that they affect the electrostatic potential landscape, particularly inside pockets.

Electromagnetic forces are the only forces acting at a molecular level and we are seeking to approach protein-protein interactions from a non-dynamical perspective; therefore, electrostatic forces are the main forces that should be taken into account. In fact, this is crudely the approach traditionally taken by chemists and biochemists when they focus their attention on the molecules' polarization or on hydrophobic or hydrophilic effects, which are basically the manifestation of electrostatic forces. We are dealing with the same issue, but in a more general manner. Furthermore, we are attempting to study not only the effects that a punctual change has on protein-protein interactions (e.g. polymorphisms) but to investigate also the possible non-local effects that such variations have on the overall interaction.

Generally, this type of systems have been studied from the mechanical classical point of view, where atomic interactions are defined *a priori* by models that do not contemplate the destruction or creation of new interactions (bonds, different types of hydrogen bonds, etc.). These classical mechanical models do not allow estimating partial atomic charges but instead require them to be assigned *a priori*. Furthermore, these charges do not vary during geometry optimization and are used for calculating the molecular electrostatic potentials (classic Poisson-Boltzmann model) [17]. We expect that quantum calculations will allow for a more precise estimation of electrostatic properties such as atomic partial charges and the electrostatic potential by modeling the electronic distribution, and that variations in these properties somehow correlated with the peptide and MHC molecule binding and recognition ability.

We developed a methodology for studying interactions between MHC-II molecules and antigenic peptides [18]–[22], which has been oriented to determining the type of electrostatic interactions present among HLA-DRβ1* molecules and different types of binding peptides. These studies have employed molecular geometries available from the Protein Data Bank (PDB) for several complexes such as: HLA-DRβ1*0101-Influenza Virus Hemaglutinine A (HA) peptide (DR1-HA, PDB code:1dlh); HLA-DRβ1*0401 Influenza Virus Hemaglutinine A peptide (DR4-HA, PDB code: 1j8h); HLA-DRβ1*0401 Collagen peptide, residues 1168–1179 (DR4-Col II, PDB code:2seb); HLA-DRβ1*0301- CLass II associated Invariant chain Peptide residues 87–101 (DR3-CLIP, PDB code:1a6ax).

These studies have focused on analyzing variations in the shape and intensity of the molecular electrostatic potential (MEP) within each pocket due to changes in the occupying amino acid, by comparing the first three terms in the multipolar expansion of the electrostatic potential, namely the monopole (*q*), the dipole moment (*d*) and the quadrupole moment (*C*), which allows ordering pockets according to their importance in the molecular interaction determining the key pockets in the HLA-DR – peptide bonding. The tools developed in these studies allowed classifying amino acids according to their electrostatic behavior into amino acids crucial for peptide's anchoring into the PBR, named *global effect* amino acids, and those responsible for a specific recognition (or weak anchoring) called *differential effect* amino acids [21].

Accordingly and in order to continue with these works, this study analyzes the HLA-DRβ1*1501 together with a Myelin Basic Protein (MBP) peptide (residues 85 to 99) (PDB code: 1BX2), named DRβ1*1501-MBP complex, by employing this methodology to further understand the main characteristics ruling peptide's bonding, find amino acids involved in the interaction within each pocket and identify a specific and reduced set of amino acids presenting high affinity in each pocket for this allele and comparing this allele with previously studied. Such methodology combined with some new approaches, seeks to define the major electrostatic interactions ruling the MHC - peptide complex formation and apply them into a more accurate and fast methodology for designing vaccines against some of the most scourging human kind diseases such as malaria.

## Materials and Methods

The molecular coordinates corresponding to the different complexes being studied in this work, were obtained from the PDB file corresponding to the HLA-DRβ1*1501-MBP complex (code: 1BX2) [23]. Several regions were defined in the HLA-DRβ1*1501 molecule according to the broadly used “pockets” approach [15], focusing our analysis on P1, P4, P7 and P9. P6 was not included in the analysis because it has been clearly shown by experimental data that there is very limited available space in this area to be considered as a pocket, due to the presence of the large size of the polymorphic β13R which hampers the conserved and critical α62N residue from establishing H bonds with the peptide's backbone [23].

Those amino acids located in a radius of 10Å around the occupying amino acid were considered to define a particular pocket [21] and are shown together with their corresponding MBP associated-fragment in Table 1 and Fig. 1B. The program VMD was used for editing the original PDB file [24]. Hydrogens and occupying amino acids were placed using Insight II (Accelrys Software, USA).

The occupying amino acid in each pocket was successively replaced by each one of the remaining 19 amino acids in order to monitor the changes in the electrostatic potential behavior induced by these replacements, keeping constant the nuclear geometry of the pockets. In this way, we built 84 molecular systems, twenty complexes for each pocket and four empty pockets, taking these later ones as the reference for each system.

The geometry of the side chain of the occupying amino acids was relaxed using classical molecular mechanics AMBER95 [25], followed by an optimization using the semi-empirical method AM1 [26]. Finally, *ab-initio* single point calculation using HF//3-21G* with Gaussian98 [27] were carried out in order to estimate the wave function and compute the Mulliken partial atomic charges [28].

Changes on the electrostatic landscape of the pockets were described by two quantities based on the three first terms of the multipolar expansion of electrostatic potential, calculated for each amino acid [21]. The charge was calculated adding the partial atomic charges of the amino acid, calculated within the Mulliken [28] approach as implemented in Gaussian98 [27]. The dipole moment vector and the quadrupole moment tensor were calculated from the atomic partial charges, using the α carbon as spatial origin (See eq. 1 to 3):
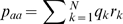
(1)

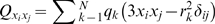
(2)

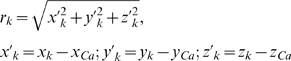
(3)where *r* is the nuclei position vector relative to the residue's α carbon and *N* is the total number of atoms in the side chain. To combine these quantities in an adimensional expression, the dipole and quadrupole magnitudes 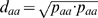 and 
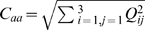
, as well as charge; were normalized according to:
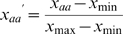
(4)


From these results descriptors for the electrostatic landscape were designed [21]. The first one named *S_aa_*:
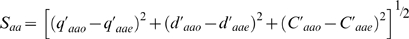
(5)where *q′* is the normalized charge of the *aa-th* amino acid in an occupied (subscript *aao*) or in an empty (subscript *aae*) pocket,; *d* ***′*** and *C* ***′*** are the dipole and quadrupole normalized moments for the respective amino acid.

The second defined quantity is a general measure for each pocket encompassing the global effect on the electrostatic landscape defined by summing *S_aa_* over all residues in the pocket:
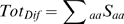
(6)


## Results

**Figure 2:**
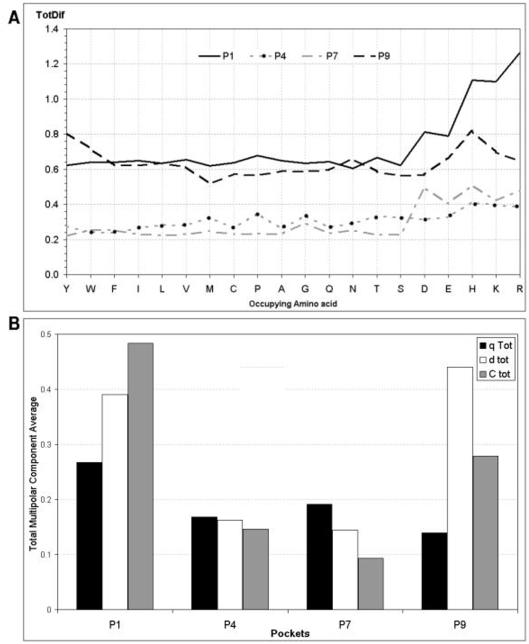
Electrostatic landscape variations according to the *Tot_Dif_*. (A) The magnitude of the total difference (*Tot_Dif_*) variable calculated for each of the pockets and with each of the occupying amino acids is shown. Large variations resulting from including each of the charged occupying amino acids into each pocket are evidenced. The magnitude of the electrostatic variation was higher for P1 and P9 compared to P4 and P7 in any system. (B) Cumulative variation of each multipolar moment where *q Tot*, *d tot* and *C tot* is the mean of total variations in multipolar moments for each pocket. The cumulative variation for Pocket 1 was high in all three multipolar moments although variation in the quadruple moment was the most prominent, while the dipolar electrostatic variation is the most notable in Pocket 9.

The *Tot_Dif_* values for each pocket with every possible occupying amino acid are shown in Fig. 2A. The average values for *Tot_Dif_* allow us to order pockets according to the magnitude of the change in the electrostatic potential landscape induced by the peptide fragment. The resulting order is P1>P9≫P4≈P7, which correlated with the pockets ability for anchoring the peptide [19], [21]. This is in agreement with experimental results [29]–[31]. It is worth mentioning that charged occupying amino acids have higher *Tot_Dif_* values than non-charged occupying amino acids, which accounts in particular for the difference between P1 and P9.

The contribution of each multipolar component to the electrostatic variation of each pocket is illustrated in Fig. 2B, showing that: (i) the quadrupole moment component has a major contribution in P1, which could account by the presence of a large number of aromatic amino acids; (ii) all three polar moments have an even contribution in P4's variations, each one being lower than polar moments in P1; (iii) P7 resembles P4's variation with slightly higher contribution of the monopole moment and (iv) a dipolar moment prevails in P9, thus evidencing the polar tendency of this pocket.

### Critical residues in MHC II – peptide interaction

The analysis of the distribution of the *S_aa_* variable allowed identifying particular electrostatic variations for each amino acid within pocket, distinguishing two types of behavior, namely *anchoring* amino acids and *recognition* amino acids.

**Figure 3:**
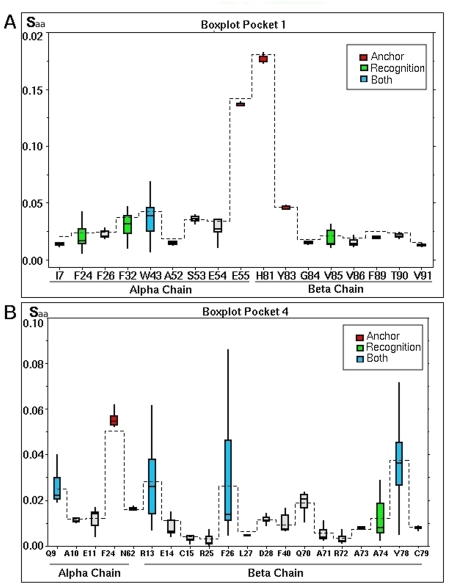
Boxplot analysis of *S_aa_* for each amino acid defining a pocket. The x-axis shows amino acids according to their position in the α and β-chain. The *mean value* of each data series are joined by a dashed line. The *median value* is the line into the box. Amino acids are classified according to their electrostatic effect which is represented by different colors: anchoring amino acids are shown in red; amino acids having a recognition effect in blue, amino acids having a dual effect are shown in green whereas those not having a notable electrostatic variation are shown in gray. The box plot analysis of Pocket 1 is illustrated in panel (A) while the analysis for Pocket 4 is illustrated in (B). The same analysis was performed for the remaining pockets (data not shown).

Such analyses were performed through the use of box plot representations, improving the methodology used by Cárdenas *et al.* [21]. This data representation does not require any assumption about the underlying statistical distribution and is based in the quartile division of the data. Thus, the first quartile (Q1) cuts off the lowest 25% of data, the second quartile (Q2), corresponding to the median, cuts the data set in half and the third quartile (Q3) cuts off the highest 25% of data, or lowest 75%. The difference between Q1 and Q3 is called the Inter Quartile Range (IQR), and is drawn as a box, whose width represents the degree of dispersion. Any data observation lying more than 1.5 IQR below Q1 or 1.5 IQR above Q3 is considered an outlier. The smallest value that is not an outlier is indicated by connecting it to the box with a line or “lower whisker”, while the largest value that is not an outlier is connected to the box by an “upper whisker” (Fig. 3) [32].

### Anchoring amino acids

A high mean *S_aa_* value indicates a high disturbance of the electrostatic potentials on this amino acid due to the presences of the peptide fragment. If it comes together with a low dispersion (small IQR) it means that the identity of the occupying amino acid is not relevant. Amino acids presenting such behavior are considered *anchoring* amino acids. Such behavior is illustrated in Figure 3A, where each box represents the distribution of *S_aa_* values together with the mean and median. It may be seen that α55E and β81H amino acids have high *S_aa_* values as well as a small box size.

**Figure 4:**
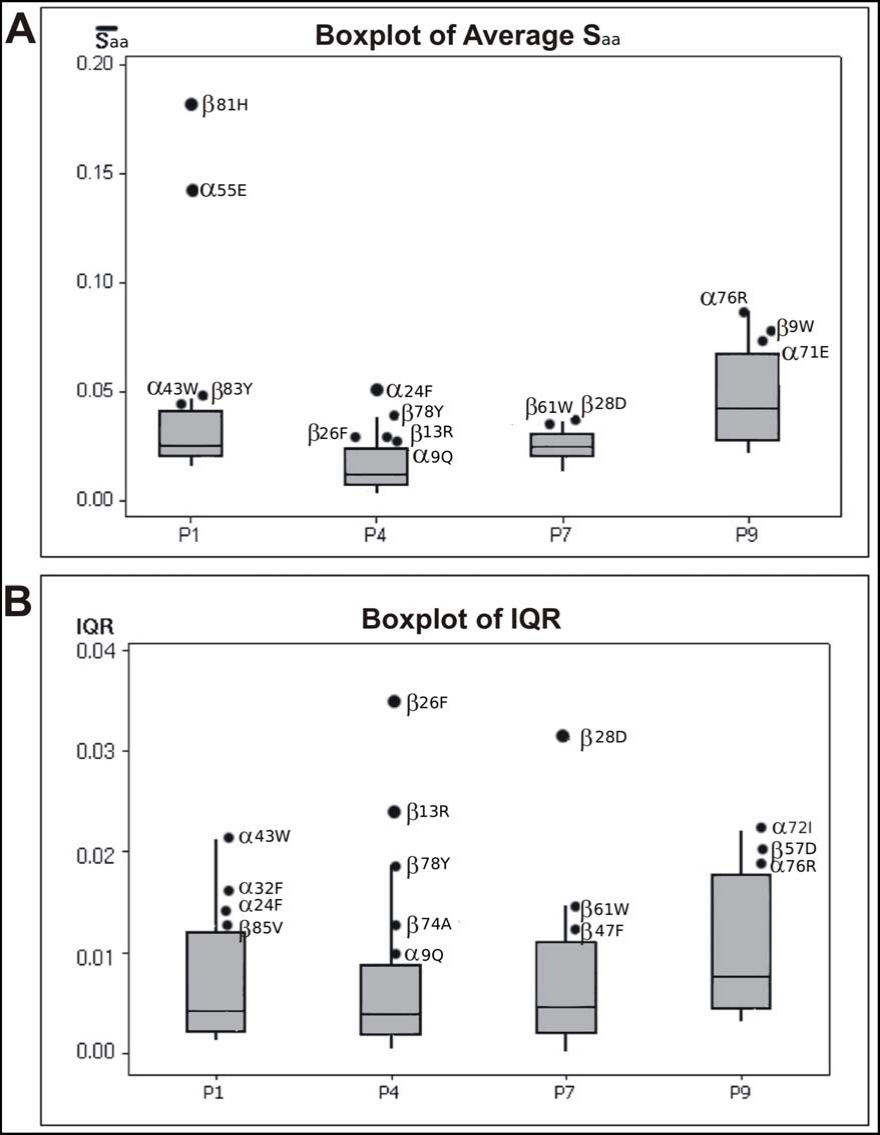
Box plot analysis applied to distinguish amino acids according to their particular electrostatic behavior. The box plot analysis allows separating data into high and low values in a quantitative-manner. High values were those lying above the third quartile and were further differentiated into outliers and upper-whisker values. (A) Summary of the *S_aa_* mean values of amino acids defining the four pockets allowing to differentiate amino acids having *S_aa_* outliers values, denoted as *primary anchoring* amino acids and those having upper-whisker values, named *secondary anchoring* amino acids. The higher anchoring effect occurred in α81H and β55E of pocket 1 (B) Summary of IQR values obtained from the box plot analysis of *S_aa_* allowing differentiating amino acids having IQR outliers values, denoted as *fine differentiation* amino acids and those having upper-whisker values, named *coarse differentiation* amino acids. The higher differentiation effect was seen in α26F, α13R and α28D residues.

In order to define whether a *S_aa_* value was high, the mean values of all amino acids defining a pocket were compared by applying a new box plot analysis. Those having values lying above the box are considered as high values. This is more precise than the former treatment of Cárdenas et al., [21]. The high values thus obtained can be further classified depending on whether they lie in the upper whisker or correspond to an outlier, the formers being denominated as *secondary anchoring* amino acids and the later ones as *primary anchoring* amino acids (Fig. 4A). The results of this analysis for the four pockets are summarized in Table 2, from which it can be seen that amino acids having a primary anchoring are buried into P1 and P4. These results agree with experimental studies revealing the importance of these pockets in peptide's binding to HLA-DRβ1*1501 molecules [30], [31], which considered the mutation over each of the peptide amino acids by Alanine and allowed the identification of peptides residues critical for binding. By the same way, our analysis shows that positions P6 and P9 are also important for peptide anchoring, although in a smaller degree, which also agrees with the results of experimental assays [30], [31].

**Table 2:** Amino acids in each pocket being critical for peptide-HLA groove interaction according to their electrostatic behavior.

Effect		P1	P4	P7	P9
**Anchoring**	**Primary**	α55E	α24F	-	-
		β81H			
	**Secondary**		α9Q		α71E
		α43W	**β13R** *	**β28D**	α76R
		β83Y	**β26F**	β61W	**β9W**
			β78Y		
**Recognition**	**Fine**	-	**β13R**	**β28D**	-
			**β26F**		
	**Coarse**	α24F	α9Q		α72I
		α32F	**β74A**	**β47F**	α76R
		α43W	β78Y	β61W	**β57D**
		β85V			

* Polymorphic positions are shown in bold-types.

Regarding secondary anchoring amino acids, they appear to be buried deep into the pockets and to be distributed along the binding groove (Table 2 and Fig. 1).

### Recognition amino acids

A high *S_aa_* dispersion value for a pocket's amino acid indicates that this is sensitive to variations in electrostatic potential resulting from the changes of the occupying amino acid; therefore it differentiates among various peptides and may be called *recognition* amino acid. In order to identify these amino acids, the dispersion of *S_aa_* was analyzed in terms of the IQR value. For instance, β13R and β26F appear to have higher dispersion IQR values in Figure 3B.

Similarly to the treatment applied to mean *S_aa_* values, a boxplot of IQRs was done allowing to identify two types of behavior for recognition amino acids: *fine recognition* (upper outliners) and *coarse recognition* (the ones lying within upper whiskers) (Fig. 4B).

As seen in Table 2, those amino acids showing a fine recognition effect are located in P4 and P7, which encompass the central region of the PBR and thereby support the selectivity role propose for these pockets [23], [30], while coarse recognition amino acids are distributed among all pockets in a similar way as secondary anchoring amino acids.

**Figure 5:**
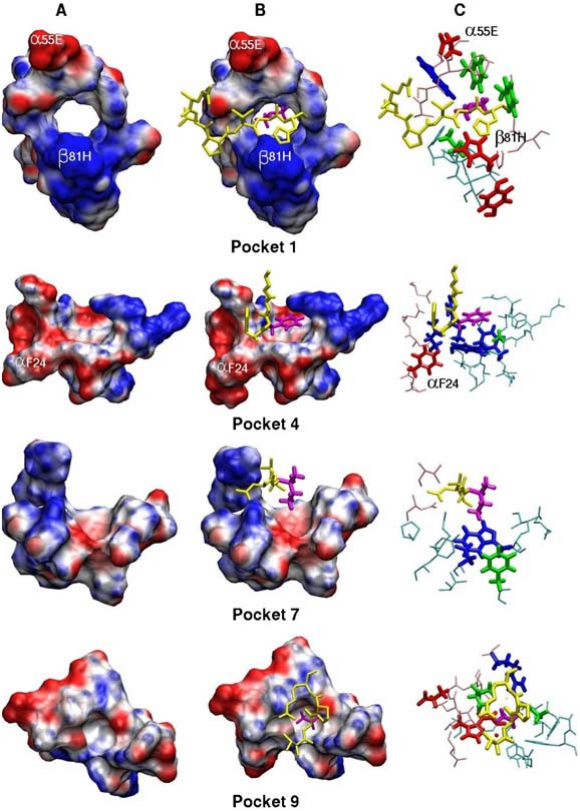
3D Pocket representations. Maximal Speed Molecular Surface representations (www.scripps.edu/~sanner/html/msms_home.html) [37] of each of the four pockets showing the position of primary anchoring amino acids within pocket 1 and 9, while amino acids indicated in pocket 4 and 7 correspond to amino acids being assign a recognition electrostatic effect (A,B). The antigenic fragment fitting into the pocket is shown in yellow sticks with the occupying amino acid highlighted in purple. (A) Empty pockets. (B) Occupied pocket. (C) Stick representation showing the disposition of anchoring amino acids (in red), recognition amino acids (in green) and amino acids having a dual effect are shown (in blue) inside pockets buried inside the PBR formed by residues lying in the α-chain (shown in pink) and the β-chain (light blue). It can clearly seen that the α55E and β81H primary anchoring amino acids of P1 are located towards the outermost portion of the binding groove (see also Fig. 1) and that α24F lies in the farthest portion of the pocket near to P1.

Figure 5 contains representations of each of the four pockets, with the disposition of primary anchoring amino acids indicated in white characters. The antigenic peptide fragment fitting into the pocket is shown in yellow sticks highlighting the occupying amino acid in purple sticks. Anchoring amino acids are shown in red and blue sticks, recognition amino acids in green and amino acids with dual effect are in blue. It can be clearly seen that the α55E and β81H primary anchoring amino acids of P1 are located towards the outermost part of the binding groove. Considering P4 it is a striking than anchoring α24F lies in the farthest portion of the pocket close to P1 (see also Fig. 1).

### Critical amino acids for each pocket

**Figure 6:**
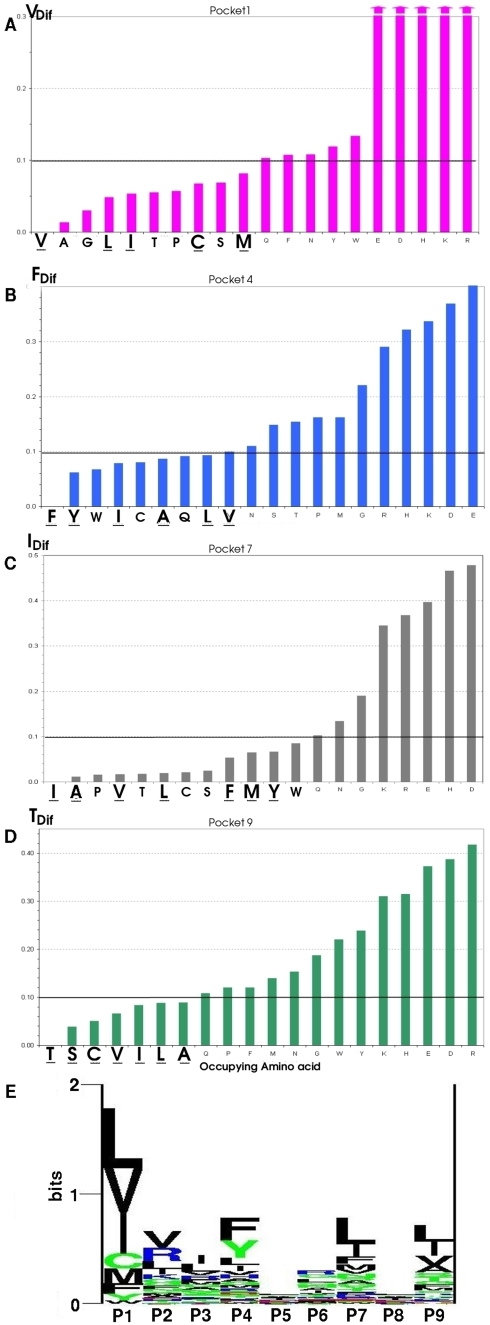
Occupying amino acid comparison with reference amino acid in MBP. Comparison of the *Ref_Dif_* between each occupying amino acid and the native occupying amino acid in the MPB peptide (V in P1; F in P4; I in P7; T in P9) allowing to detect those amino acids more closely resembling to the reference amino acid in terms of global variations in the electrostatic landscape. (E) Logo displaying the experimentally recognized binding residues and motifs of HLA-DRβ1*1501 where the height of each column represents the information content (in bits) at the given position in the binding motif. The height of each letter within a column is proportional to the “frequency of the corresponding amino acid at that position”. The experimental data compelled in this graph was taken as reference of each pocket's preferential binding ability [33]. Download: www.cbs.dtu.dk/researchgroups/immunology/supertypes.php. Amino acids experimentally proven to have a preferential binding in each pocket according to the logo data are underlined in figures B, C, D and E, indicating that the theoretical analysis is in high agreement with the experimental data.

Up to here we have compared the changes in electrostatic landscape in occupied pockets against the empty pocket in a further step the change against the native amino acid may be compared by means of the following expression:
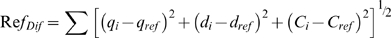
(7)where the subscript *ref* refers to the amino acid in the occupied pocket by the reference or native amino acid (V in P1; F in P4; I in P7; T in P9 in the MBP) and *i* refers to the replaced amino acid on the pocket. These *Ref_Dif_* value for each occupying amino acid allow us to detect those amino acid more closely resembling the native ones in terms of global variations of the electrostatic landscape (Fig. 6).

For P1, the aliphatic amino acids A, G, L and I closely resemble the reference amino acid (V) followed by T, P, C, S, M and F (Fig. 6A). The underlined amino acids correspond to those which have been experimentally determined to be occupying this pocket in a large number of peptides binding with high affinity to HLA-DRβ1*1501 purified molecules (Fig. 6E) [33], [34].

The reference amino acid in P4 is phenylalanine (F). Aromatic Y and W closely resemble in first place; and in second place aliphatic apolar amino acids: I, C, A, L, M and the only polar Q can replace F (Fig. 6B). The allelic variation in the β71A position enables the entrance of larger occupying amino acids into this pocket [23].

Regarding P7, originally occupied by an isoleucine (I), it is found that the closest amino acids are mainly the group of varying-size aliphatic amino acids: A, P, V and L; followed by a second group of small polar amino acids such as T, C, S and by third group including the aromatic amino acids F, Y, W (Fig. 6C). The principal experimental binding amino acids L, I, F and V for this allele (Fig. 6E), are in the identified set of amino acids.

Finally for P9, S, A, C, I, V and L were found as being the more closely resembling the reference occupying (Threonine) (Fig. 6D), in complete agreement with those experimentally reported (Fig. 6E).V and L, are small-sized aliphatic amino acids as well as the small polar T, C and S amino acids. This is in agreement to the electrostatic and sterical characteristics found for this pocket [21].

All these results reveal great agreement between our data obtained by quantum chemistry and those obtained experimentally.

## Discussion

In all complexes studied so far, P1 stands always in the first place of importance regarding variation in the electrostatic potential landscape. The genetic region encoding amino acids defining this pocket is largely conserved within the different alleles studied, presenting just a single dimorphic variation in the Gβ86V position among human HLA-DRβ1* molecules. Such genetic conservation allows for the electrostatic potential to be maintained in all peptide - MHC II interactions, thereby ensuring that the anchoring characteristics assigned to this pocket are preserved, as clearly demonstrated by studies with truncated antigenic peptides showing that peptide binding to any MHC II molecule diminish considerably when the peptide's N-terminal is cleaved [30], [31].

The next pocket in the proposed order is P9, with a variation in electrostatic landscape not far from P1. Experimentally it has been found that P9's depth and low polymorphism contribute to the stabilization peptide's anchoring to Class II molecules.

On the other hand, allelic and anchoring specificity is given by peptides in pockets 4 and 7 (or 6 in other cases) determined by their considerable degree of polymorphism.

### Defining the characteristics of each Pocket

A general analysis regarding variations of the electrostatic landscape in terms of *S_aa_* and *Tot_Dif_* allow defining the particular electrostatic character of each pocket and the characteristics of its interaction with the occupying peptide.

#### Pocket 1

Given that this is the most important pocket for antigenic peptides binding, it contains two primary anchoring amino acids: β81H and α55E; the former enables the formation of hydrogen bonds with the peptide's backbone and is essential in binding peptides to the MHC II molecule [35]. These two primary anchoring amino acids are located in the pocket's outermost region towards the end of the PBR (Fig. 1B and 5A, B, C) and have an independent electrostatic behavior regardless of the allele they belong to since the genetic region encoding this pocket is genetically conserved [20], [21]. Fine recognition amino acids were not identified in this pocket, thus supporting the statement that this pocket is mainly involved in the anchorage of peptides.

Coarse recognition amino acids (α43W, α32F and α24F) as well as secondary anchoring α43W identified for P1 are all aromatic (Fig. 4), thereby accounting for a high disturbance in the quadrupole component of this pocket. Such notable characteristic should be somehow related to the fact that L, V and I are the prominent amino acids fitting into this pocket as shown in Figure 6E [33], totally agreeing with our results (Fig. 6A). It is worth stressing that the allelic variation Vβ86G of this pocket accounts for an increased affinity of smaller amino acids in this pocket compared to the size of amino acids fitting into HLA-DRβ1*0101 and HLA-DRβ1*0401 alleles (mainly the aromatic amino acids W, Y and F) [19], [33].

The DRβ*W2901-05, W609 allele analogues in *Aotus* monkeys bear the Fβ86V polymorphism, which allows supposing that the small aliphatic amino acids A, G, S, T and P, found in the electrostatic analysis, fit into Pocket 1. Nevertheless this same allelic variation in humans has only been found in the HLA-DRβ1*0805 molecule [36].

#### Pocket 4

The binding of F to the primarily hydrophobic P4 is a prominent feature of HLA-DRβ1*1501 (Fig. B, E) facilitated by the aromatic residues α24F, β78Y and β26F, perhaps contributing to binding of aromatic amino acids such as F and Y [23]. This is in accord with our findings. It is worth highlighting that the first two positions are conserved among the different HLADRβ1*1501 variants whereas β26F is polymorphic. We found that an important polymorphic position is β13R because it has both fine recognition and high anchoring effects. It seems that this is the important and typical position in this allele. On our side, β71A polymorphism in this pocket seems to be important for creating an appropriate space for accommodating the lateral chains of aromatic amino acids [23]. Most HLA-DR alleles bear K, R or E in the β71 position, which is why most amino acids buried into this pocket have a small size and are mostly polar. Therefore the presence of the β71A polymorphic substitution in the HLA-DR1*1501 molecule represents a prominent aspect of this allele specificity for anchoring peptides, which is practically exclusive of HLA-DR1*1501 variants [10]. Polymorphic β71A position causes a steric effect which could not perceive in our electrostatic study.

#### Pocket 7

The P7 is characterized by a mixed polar-aromatic character due to three relevant amino acids: β28D, β61W and β47F. The former two are mixed in character and the third one is a coarse recognition amino acid (Fig. 4A). Besides, it should be noted that the β28D position is polymorphic, rendering it crucial in this pocket's selectivity (Fig. 4B). None primary anchoring amino acids were identified in this pocket, which allows assigning it a mainly recognition function (Fig. 4).

#### Pocket 9

A differential electrostatic behavior was evidenced in P9 when compared to P9 in all other studied alleles. This cannot be explained only by the few polymorphic differences in β9, β30 and β37 positions among the different alleles [19], [22]. Our results report a global change in the electrostatic variation in P9 belonging to the HLA-DRβ1*1501 molecule and neither anchoring primary nor fine recognition amino acids were identified, since all amino acids defining this pocket exhibit an even electrostatic behavior (Table 2), indicating that no amino acids is affected specifically by the presence of the occupying amino acid (in Fig. 4 are not outliers). Experimental analyses have shown that peptide can have a binding affinity with HLA-DRβ1*1501, even if, the peptide does not have amino acid in P9 and far away, so the interaction with this pocket has been suggested that is with the backbone [30], our results are correlated with this observation.

The electrostatic variation seen in this pocket's dipolar moment could be understood by the polar character of the α71E, α76R and β57D contained in this pocket (Fig. 2B).

A comparison of the electrostatic variations in this pocket against the reference occupying amino acid T revealed a pair of polar (S and C) amino acids and a reduced set of aliphatic amino acids (V, I and L) showing a similar behavior. The Wβ9E and Wβ9Q polymorphic variations in this secondary anchoring amino acid is believed to be responsible for the preference of large polar occupying amino acids such as R, H and K in P9 in allele molecules bearing this polymorphism, as occurs in HLA-DRβ1*0401, 0402, 0404 and in HLA-DRβ1*1501 allelic forms named HLA-DRβ5*02 [36].

The function of some relevant amino acids in the overall interaction should be highlighted due to their presence in different pockets, or for exhibiting anchoring and recognition characteristics in the same pocket simultaneously. Among the amino acids presenting such behavior are: α24F, α76R, β78Y, β61W, β13R and β28D, of which the first four positions are conserved whereas the remaining are polymorphic and possibly account for the selectivity of this allele. Our results have shown that such positions as well as some other polymorphic positions are important both in recognition and anchoring effects, having profound implications in the specificity of this allele.

Other characteristic of the effects in this allele is that primary anchoring is observed in conserved position located in P1 and P4 similar to others alleles, in contrast to fine recognition which is observed in polymorphic positions (P4 and P7). Therefore, a peptide might be binding in whatever allele, but the final recognition is done by polymorphic amino acids.

In summary, the overall electrostatic variation in each pocket due to the presence of each of the 20 possible occupying peptides (see Figure 2A) reveals a differential electrostatic effect in each of the binding pockets, which could be ordered according to the magnitude of the electrostatic variation in decreasing order as follows: P1>P9≫P4≈P7, this order correlates well with the ordering experimentally found for anchorage of the antigenic peptide.

An overall analysis of the system's behavior leads to conclude that P1 and P4 are mainly involved in the peptide's anchorage, in agreement with experimental studies [29], [30]. Furthermore, such anchorage could be attributed to the α55E, β81H and α24F conserved amino acids particularly. The same experimental studies have reported that the recognition effects occur inside the binding groove, mainly in the region spanning between P4 and P7, where the three remaining β13R, β26F, β28D polymorphic amino acids were found by us.

In contrast to other pockets, P9 exhibits a notable anchoring and recognition effect which can not be attributed to any particular amino acid defining this pocket, since a rather even contribution was evidenced in these amino acids.

It is worth noting that the methodology here proposed for analyzing the MCH II – peptide interaction in terms of the two electrostatic molecular descriptors permits to identify the reduced set of amino acids important for such interaction in a particular allele, especially those amino acids in polymorphic positions that should be considered when designing synthetic peptides capable of interacting specifically with this allele, seeking to accomplish a rational, logic, design of synthetic, multi-epitopic and allele-specific vaccine based on the quantum chemical behavior of proteins.
